# Mutation in *MEOX1* gene causes a recessive Klippel-Feil syndrome subtype

**DOI:** 10.1186/1471-2156-14-95

**Published:** 2013-09-28

**Authors:** Fatih Bayrakli, Bulent Guclu, Cengiz Yakicier, Hatice Balaban, Ugur Kartal, Bekir Erguner, Mahmut Samil Sagiroglu, Sirin Yuksel, Ahmet Rasit Ozturk, Burak Kazanci, Unal Ozum, Hamit Zafer Kars

**Affiliations:** 1 Department of Neurosurgery, Cumhuriyet University School of Medicine, Kampus, Merkez, 58140, Sivas, Turkey; 2 Molecular Neurogenetics Research Laboratory, Department of Neurosurgery, Cumhuriyet University School of Medicine, Sivas, Turkey; 3 Neurosurgery Clinic, Ministry of Health, Sevket Yilmaz Research and Training Hospital, Bursa, Turkey; 4 Department of Medical Biology, Acibadem University School of Medicine, İstanbul, Turkey; 5 Department of Neurology, Cumhuriyet University School of Medicine, Sivas, Turkey; 6 TUBITAK BILGEM, Kocaeli, Turkey; 7 Informatics Institute, Middle East Technical University, Ankara, Turkey

**Keywords:** Klippel-Feil syndrome, MEOX1, Whole-exome sequencing, Vertebra, Whole genome linkage analysis

## Abstract

**Background:**

Klippel-Feil syndrome (KFS) is characterized by the developmental failure of the cervical spine and has two dominantly inherited subtypes. Affected individuals who are the children of a consanguineous marriage are extremely rare in the medical literature, but the gene responsible for this recessive trait subtype of KFS has recently been reported.

**Results:**

We identified a family with the KFS phenotype in which their parents have a consanguineous marriage. Radiological examinations revealed that they carry fusion defects and numerical abnormalities in the cervical spine, scoliosis, malformations of the cranial base, and Sprengel’s deformity. We applied whole genome linkage and whole-exome sequencing analysis to identify the chromosomal locus and gene mutated in this family. Whole genome linkage analysis revealed a significant linkage to chromosome *17q12*-*q33* with a LOD score of 4.2. Exome sequencing identified the G > A p.Q84X mutation in the *MEOX1* gene, which is segregated based on pedigree status. Homozygous *MEOX1* mutations have reportedly caused a similar phenotype in knockout mice.

**Conclusions:**

Here, we report a truncating mutation in the *MEOX1* gene in a KFS family with an autosomal recessive trait. Together with another recently reported study and the knockout mouse model, our results suggest that mutations in *MEOX1* cause a recessive KFS phenotype in humans.

## Background

Klippel-Feil syndrome (KFS) is clinically characterized by a short neck, low posterior hairline, and limited neck movement. These abnormalities result from the failure of the cervical vertebrae to correctly segment during embryonic development; there might also be associated anomalies in different organ systems [1,2]. Sprengel’s deformity, which sometimes accompanies KFS, is the congenital elevation of the scapula, in which approximately 50% of cases have an omovertebral bone [3].

Genetically, KFS has two subtypes with an autosomal dominant trait: KFS1 (OMIM #118100) is caused by mutations in the GDF6 gene, while KFS3 (OMIM #613702) harbors mutations in the GDF3 gene. An autosomal recessive subtype, KFS2 (OMIM%214300), also exists, in which the causative gene has recently been reported. In this paper, we report on our finding that MEOX1, located on chromosome 17q21.31, is the causative gene of KFS2 as revealed in a Turkish family with the autosomal recessive KFS trait.

## Results and discussion

**Figure 1:**
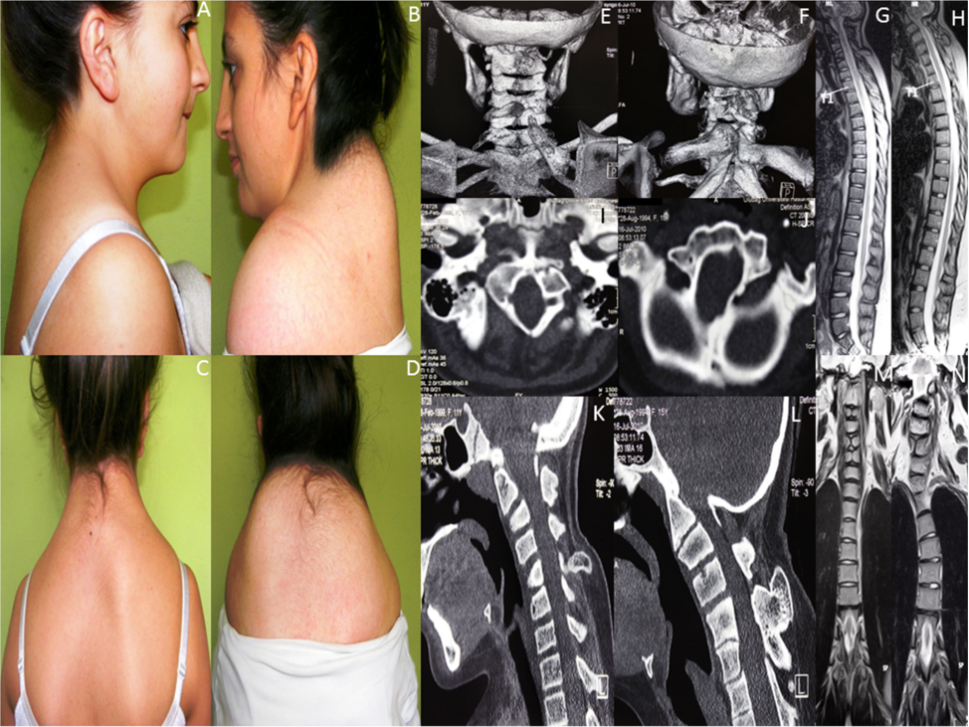
**Patient photographs of family members IV-2 and IV-3 show KFS phenotypes.** Photographs of patients from the side **(A** and **B)** and behind **(C** and **D)** show short neck, low posterior hairline, and elevated scapula (Sprengel’s deformity). 3D cervical spine skull base CT images show omovertebral bone causing Sprengel’s deformity, posterior neural arch fusion defects, and fusions between vertebrae **(E** and **F)**. Whole spine sagittal section T2-weighted MR images show cervical vertebrae count anomaly **(G** and **H)**. Axial skull base CT images show malformation of the foramen magnum **(I** and **J)**. Sagittal reconstruction of cervical and skull base CT images show abnormal bony formation emerging from posterior structures as well as fusion between posterior structures of some successive vertebrae, occipitalization of atlas and dens, and corpus anomalies **(K** and **L)**. Scoliosis is evident on coronal T2-weighted whole spine MR images. Fusion defects are seen in midportions of vertebra corpuses **(M** and **N)**.

Physical findings for the parents and unaffected members of the family were normal. All seven affected family members had a short neck, low posterior hairline, limited neck movements, and elevated scapula (Figure 1). Computerized tomography (CT) with sagittal and 3D reconstruction of the skull base and cervical spine and whole spine magnetic resonance imaging (MRI) of sagittal and coronal sections of affected individuals revealed a structural and configurational anomaly of the foramen magnum, occipitalization of the atlas (C1 vertebrae), fusion between the atlas and axis (C2 vertebrae), fusion between the anterior and posterior structures of some cervical vertebrae, a short anteroposterior diameter of several cervical vertebral corpora (bodies), and omovertebral bone between the scapula and posterior structures of cervical vertebrae causing Sprengel’s deformity. In addition, neural arch fusion defects occurred at some cervical levels (posterior fusion defects). In coronal sections of whole spine MRI, scoliosis was evident and sagittal sections revealed a numerical decrease in the cervical vertebrae count as a result of fusion between them (Figure 1). There was no structural anomaly of the brain tissue.

**Figure 2:**
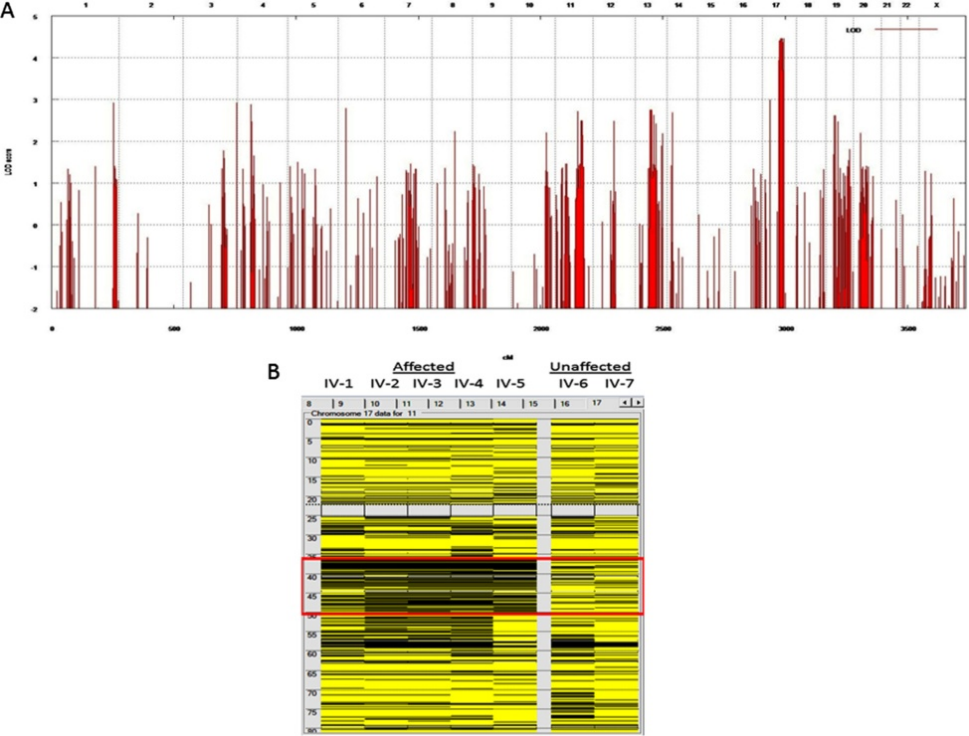
**Genome-wide linkage analysis with and homozygosity mapping 250 K Nsp I Affymetrix Gene Chips. (A)** the locus on chromosome 17 was the only one to give the theoretical maximum for this pedigree. cM, centimorgan; LOD, logarithm of odds. **(B)** homozygosity mapping of KFS family via autoSNPa. Each column represents the homozygosity profile of one individual, and each row represents the call of an individual SNP on chromosome 17 (yellow, heterozygous; black, homozygous). Note the large run of homozygosity that is exclusively shared by the affected members (boxed in red).

Family members III-1, III-2, IV-1, IV-2, IV-3, IV-5, IV-6, and IV-7 were analyzed by genome-wide linkage analysis and a theoretic maximum logarithm of the odds (LOD) score of 4.2 was identified at chromosome 17q12-33 between genetic markers rs8066255 and rs2958872 (Figure 2A). This locus was the only site with a LOD score above 3 and, was found to contain 16 Mb and nearly 200 genes. Using genotype data of the same family members, homozygous mapping showed the same chromosomal locus to be shared by all affected individuals (Figure 2B).

Genomic DNA samples from family members III-2, IV-1, IV-2, IV-3, IV-4, IV-5, IV-6, and IV-7 were analyzed by whole-exome sequencing for causative mutations that segregated with the pedigree status. We achieved a mean coverage of 44×, and 94% of all targeted bases were read more than four times, which was sufficient to identify novel homozygous variants with high specificity. We identified mutations in six different genes within our linkage area that segregated with pedigree status. We observed a single nucleotide change GTC → GCC in the AOC3 gene that caused an amino acid substitution (V14A). AOC3 is expressed on the surface of endothelial cells and is involved in leukocyte trafficking. Homozygous null mice are healthy, fertile, and viable, but display decreased lymphocyte migration and homing in response to inflammation [4,5]. We found another single nucleotide change in the MRPL27 gene that causes an amino acid substitution (C → T, p.R74C). This mutation is located in the N terminal region of the protein. MRPL27 encodes a mitochondrial ribosomal protein, L27, and mutations in this family of genes are mainly involved in oxidative phosphorylation deficiencies [6]. The KRTAP4-11 gene was also mutated (C125Y TGC → TAC) and the encoded protein is a member of the keratin-associated protein (KAP) family, which form a matrix of keratin intermediate filaments that contribute to the structure of hair fibers. However, the observed amino acid change was not located within a highly conserved region. Mutations were also identified in the ORMDL3 gene (delCCCATCTTTCCCCAAC in the 3′ UTR), which is associated with susceptibility to asthma, and the GHDC gene (delCAC p.W281del), which has no known functional significance [7,8]. Thus, we excluded these five candidate genes as causative for the KFS phenotype. The final change was a novel homozygous nonsense mutation within the MEOX1 gene (Figure 2B).

**Figure 3:**
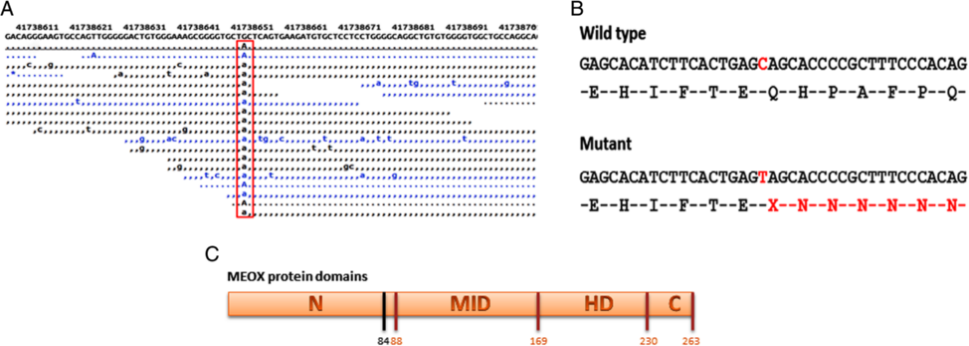
***MEOX1 *mutation analysis. (A)** G → A nucleotide change (red box) in *MEOX1* is identified by exome sequencing. **(B)** This nucleotide change causes the formation of a stop codon and a truncated protein. **(C)** The mutation is located at the end of the N terminal domain (N: N terminal domain, MID: middle domain, HD: home domain, C: C terminal domain; black vertical line shows mutation location).

**Figure 4:**
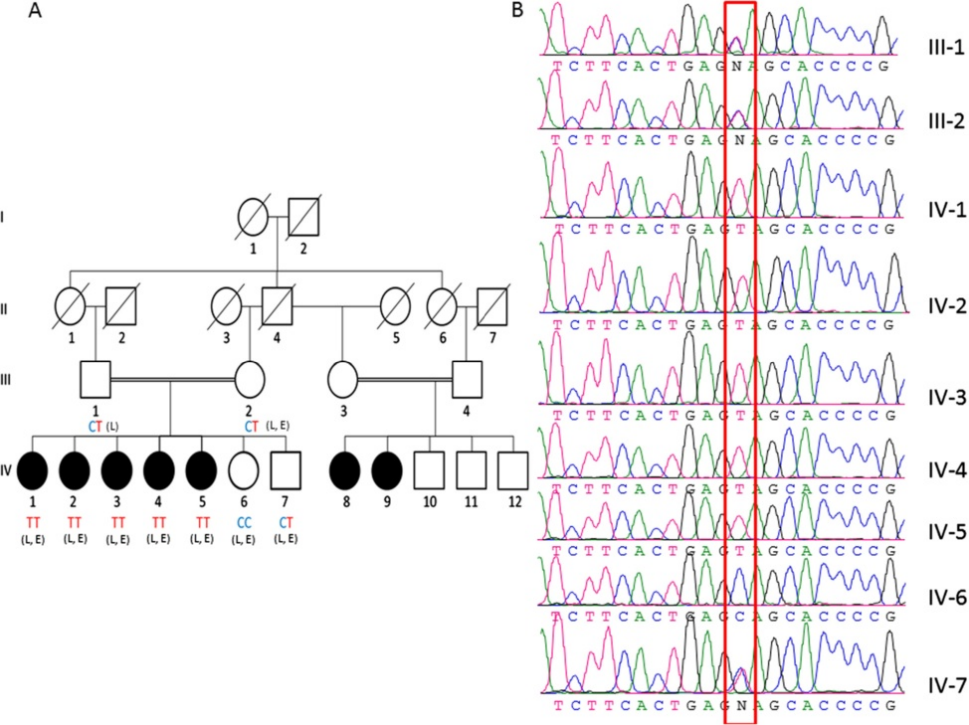
**KFS family pedigree. (A)** Affected sibs are identified by filled symbols. Diagonal lines indicate deceased family members. Circles represent female and squares represent male family members. The affected genotype is TT, healthy individuals are CT and CC genotypes. “L” represents patients used in whole genome linkage analysis; “E” represents those used in whole-exome sequencing. **(B)** Sanger sequencing confirms the altered base in family members (red box).

Full-length MEOX1 (NM_001083961) maps to chromosome 17q21.31 and encodes a 254 amino acid protein. The mutation identified by whole-exome sequencing causes a 670G → A nucleotide change in codon 84 within the first exon of MEOX1, resulting in the premature stop codon Q84X and a truncated protein (Figure 3A and B). The mutation was confirmed by Sanger sequencing to be homozygous in all affected family members and to be heterozygous in parents and in one unaffected child (Figure 4A and B). Our results therefore indicate that the homozygous mutation in MEOX1 causes autosomal recessive KFS. While this paper was in preparation, Mohamed et al. [9] identified two additional families with KFS also carrying homozygous mutations in MEOX1. Together with this report, our study is the first to describe a recessive KFS phenotype in humans and its causative mutation in the gene MEOX1.

Somitogenesis is a series of dynamic morphogenetic events that involve the cyclic signaling of different pathways, such as Notch signaling [4]. Among the many genes that regulate somitogenesis, *MEOX1* and *MEOX2* are particularly important genes [5]. *MEOX1* was initially isolated from *Mus musculus*, where it is first expressed during gastrulation in the early mesoderm and later in the presomitic mesoderm, early somites, and throughout the sclerotome and dermomyotome [5]. Mankoo et al. [6] previously showed that mouse embryos deficient for *MEOX1* and *MEOX2* form somites in a disorganized manner, have no recognizable dermomyotome and show no rostrocaudal polarization or sclerotomal segmentation. Phenotypes of these mutants also include deficiencies in skeletal muscles, and lack ribs and vertebrae. The authors therefore concluded that *MEOX1* and *MEOX2* are required for correct gene expression in all somatic compartments.

In an earlier study by Mankoo et al. [7], homozygous single null *MEOX2* mutant mice lacked specific muscles and showed a reduced muscle mass but a normal axial skeleton; the authors suggested that, in this case, *MEOX1* substitutes for *MEOX2* in the sclerotome but not the myotome. Skuntz et al. [5] studied single *MEOX1* null mutant mice and found that they have defects in the axial skeleton but not in muscle development. Thus, the authors suggested that *MEOX2* compensates for the lack of *MEOX1* in the myotome but not the sclerotome. In their study, *MEOX1* associated mutants showed major alterations in cranio-cervical joints, indicating that *MEOX1* plays important, non-redundant roles in maintaining sclerotome polarity and the formation of cranio-cervical joints. In addition, heterozygous mutant mouse phenotypes were shown to be similar to wild-type mice. Closer analysis of the phenotype the homozygous *MEOX1* mutant mice revealed that the bones of cranio-cervical joints were remodeled such that the anterior arch of the atlas was assimilated into the basioccipital bone and neural arch as well as partially deleted and/or fused basioccipitals. Moreover, the dens of the axis was deleted or projected upward or fused with the atlas. These homozygous mutant mice also showed vertebral fusions and split vertebral ossification centers.

Interestingly, the phenotype of the affected members of our family showed very similar clinical and radiological features to the homozygous mutant mice in the study by Skuntz et al. [5] reported. The mouse model showed vertebral anomalies in the entire spine, including the lumbar and sacral region. However, in our patients, the vertebral defects were limited to the cervical region. In addition to this similar phenotype, our affected human subjects had additional omovertebral bones between the scapula and low posterior cervical spine structures causing Sprengel’s deformity.

The homozygous mutation we identified in our KFS family has several lines of evidence supporting its involvement in the disease phenotype. First, similar mutations were found in a report by Mohamed et al. [8] in exons 1 and 3 of the *MEOX1* gene in two KFS families. The homozygous mutation we identified in our KFS family has several lines of evidence supporting its involvement in the disease phenotype. First, similar mutations were found in a report by Mohamed et al. [9]. This pathway is one of the control mechanisms ensuring the fidelity of gene expression in which destabilization of nonsense-containing mRNAs depends on recognition of the nonsense codon by the translational machinery [9].

MEOX1 has an N terminal, middle, and C terminal domain, as well as a home domain [10]. Our premature termination codon mutation is located in exon 1 of *MEOX1* near the end of the N terminal domain (Figure 3C). However, as Mohamed et al. showed by RT-PCR, no protein is produced despite the presence of nucleotides before the premature stop codon because of the NMD mechanism. If truncated proteins were produced, it would lack the DNA binding homebox and therefore would be expected to be a dominant negative mutation such that heterozygote carriers of the mutation would show some KFS disease phenotypes. The absence of this in heterozygous parents and unaffected siblings provides evidence against the production of truncated protein. To further test whether this mutation is disease-causing, we screened 100 unrelated individuals for this variation. None of the 200 chromosomes examined carried the mutation. Segregation analysis of the mutant *MEOX1* allele revealed that both parents and healthy siblings carried the mutation in the heterozygous state, whereas all affected family members had the homozygous mutation.

## Conclusions

The present study provides sufficient evidence that KFS in this Turkish family is caused by a mutation in the *MEOX1* gene. This corroborates the essential role of MEOX1 in maintaining sclerotome polarity and the formation of cranio-cervical joints as shown previously in transgenic experiments.

## Methods

The study was approved by the Cumhuriyet University Committee of Assessment of Scientific Research (protocol number: 2011-005). We identified a family in western Turkey diagnosed with KFS based on clinical and radiological records. Consent to participate in the study, for the publication of identifiable figures, and for the collection of blood samples was obtained from the family. DNA was then prepared using standard techniques.

### Linkage analysis and homozygosity mapping

We genotyped family members III-1, III-2, IV-1, IV-2, IV-3, IV-5, IV-6, and IV-7 using GeneChip Mapping 250 K Nsp Arrays (Affymetrix, Santa Clara, CA) for genome-wide linkage analysis. Single nucleotide polymorphism (SNP) genotypes were obtained by following the Affymetrix GeneChip Mapping protocol using the GCOS program (Affymetrix). Using the identified genome analysis programs, basic analysis and manipulation of the GeneChip data were undertaken. Multipoint Engine for Rapid Likelihood Inference (Merlin) software performed the multipoint linkage analysis [11]. We assumed autosomal recessive inheritance and assigned 99% penetrance and a phenocopy rate of 0.001. Allele frequencies for the GeneChips SNPs were obtained from Affymetrix.

Autozygosity mapping was performed on the resulting genotypes with autoSNPa (http://autozygosity.org/autosnpa) to look for shared homozygous regions by affected [12].

### Exome sequencing

Genomic DNA samples of III-2, IV-1, IV-2, IV-3, IV-4, IV-5, IV-6, and IV-7 were prepared for whole-exome sequencing using the TruSeq Sample Preparation Kit (Illumina). Exonic regions of the samples were captured using the TruSeq Exome Enrichment Kit (Illumina). The TruSeq PE Cluster Kit v3-cBot-HS was used for paired-end cluster generation and the TruSeq SBS Kit v3-HS reagent kit for sequencing post-capture libraries (Illumina). Initial clustering was performed on an Illumina cBot machine and paired-end sequencing was carried out on an Illumina HiSeq 2000 system with a read length of 104. All procedures were carried out according to the manufacturer’s instructions. Base calling and image analysis were performed using Illumina’s Real Time Analysis software version 1.13 with default parameters.

The paired-end sequence reads were aligned to the human genome (hg19) using BWA software [13]. SAMtools was used to remove PCR duplicates [14]. After duplication removal, the depth of coverage of targeted exome regions was calculated using BEDtools [15]. Furthermore, 75% of targeted regions were sequenced with at least eight-fold coverage, which is sufficient to identify both homozygous and heterozygous variants. Realignment around insertions and deletions (indels) was performed using The Genome Analysis Toolkit v1.6 (GATK) IndelRealigner [16]. To discover single nucleotide substitutions (SNSs) and small indels, the GATK Unified Genotyper was run on aligned reads with recommended parameters. Variants with a quality score lower than 50 were discarded. The remaining variants were annotated for novelty by comparing with dbSNP (build 135).

Functional annotation of variants with an adequate quality score was performed using ANNOVAR [17]. Variants of interest were then sifted using VarSifter [18] in which positions that indicated a homozygous variant in affected siblings, a heterozygous or homozygous normal variant in non-affected siblings, and a heterozygous variant in the mother were selected for further inspection. The final set of selected variants was visually inspected using Integrative Genomics Viewer (IGV) [19].

### Mutation confirmation by PCR

To confirm the mutation, polymerase chain reaction (PCR) primers covering the mutation site in *MEOX1* were designed using PRIMER3 (http://bioinfo.ut.ee/primer3-0.4.0/). A BLAST homology analysis was performed using the National Center for Biotechnology Information Web tool (http://www.ncbi.nlm.nih.gov/blast) to compare individual sequences with wild-type sequences. All *MEOX1* exons were amplified using standard PCR techniques and were directly sequenced, as previously described, for each family member.

## Competing interests

All authors declare that they have no competing interests.

## Authors’ contributions

FB: have made substantial contributions to conception and design, acquisition of data, analysis and interpretation of data; have been involved in drafting the manuscript or revising it critically for important intellectual content; have given final approval of the version to be published. BG: have made substantial contributions to conception and design, acquisition of data, analysis and interpretation of data; have been involved in drafting the manuscript or revising it critically for important intellectual content; have given final approval of the version to be published. CY: have made substantial contributions to conception and design, acquisition of data, analysis and interpretation of data; have been involved in drafting the manuscript or revising it critically for important intellectual content; have given final approval of the version to be published. HB: have been involved in drafting the manuscript, revising it critically for important intellectual content; have given final approval of the version to be published. UK: have been involved in drafting the manuscript, revising it critically for important intellectual content; have given final approval of the version to be published. BE: have made substantial contributions to conception and design, acquisition of data, analysis and interpretation of data. MSS: have made substantial contributions to conception and design, acquisition of data, analysis and interpretation of data. SY: have been involved in drafting the manuscript, revising it critically for important intellectual content; have given final approval of the version to be published. ARO: have made substantial contributions to conception and design, acquisition of data, analysis and interpretation of data. BK: has made substantial contributions to conception and design, acquisition of data, analysis and interpretation of data. UO: has made substantial contributions to conception and design, acquisition of data, analysis and interpretation of data. HZK: have been involved in drafting the manuscript, revising it critically for important intellectual content; has given final approval of the version to be published, have made substantial contributions to conception and design, acquisition of data, analysis and interpretation of data. All authors read and approved the final manuscript.
